# Ochratoxin A in Roasted Coffee from French Supermarkets and Transfer in Coffee Beverages: Comparison of Analysis Methods 

**DOI:** 10.3390/toxins2081928

**Published:** 2010-07-28

**Authors:** Mariana Tozlovanu, Annie Pfohl-Leszkowicz

**Affiliations:** Laboratory Chemical Engineering, Department Bioprocess & Microbial System, UMR CNRS/INPT/UPS 5503, ENSA Toulouse, France; Email: mariana.tozlovanu@ensat.fr (M.T.)

**Keywords:** ochratoxin, coffee, beverage, immunoaffinity column, CEN 14132, CEN 15141

## Abstract

The OTA content of 30 roasted coffees purchased in French supermarkets was evaluated by two validated different methods: one using immunoaffinity column (IAC) clean-up after alkaline extraction; the second using toluene extraction under acidic conditions. OTA recoveries (0.5 to 5 µg/kg) ranged from 16–49% with the alkaline extraction method and 55–60% with the acidic method. OTA recoveries from prepared beverages were similar with all methods (75–80%). All samples containing OTA ranged from trace (<LOQ) to 11.9 µg/kg. About 20 to 140% of OTA passed through the beverages. Recoveries of over 100% of OTA in beverages were due to three types of interferences: (i) formation of open-ring OTA (OP-OA) during alkaline extraction, (ii) isomerization of OTA during roasting, and (iii) presence of the nonchlorinated analogue OTB. The first two types of interference generate OTA derivatives that are not recognized by OTA antibodies, while OTB cross-reacts with OTA-antibodies. These analytical problems will seriously impact the amount of OTA detected, especially at the levels close to the limits from the EU legislation. Underestimation of OTA could be highly dangerous for health.

## 1. Introduction

Ochratoxin A (OTA) is a secondary metabolite produced either by *Penicillia* in cereal or *Aspergillii* in wine and coffee [1,2]. OTA is a nephrotoxic and carcinogenic compound found in several food products such as cereals, nuts, beer, and wine [3,4]. Similar to other crops, coffee fruits and beans can be contaminated by toxigenic fungi, which, besides altering the quality of coffee [5,6,7] may present a serious risk of OTA contamination, compromising the safety of the product. OTA has also been detected in green and roasted coffee beans [3,8,9,10,11,12,13]. As OTA is not significantly reduced by roasting, the final coffee brew could also be an important OTA source in the human diet [14,15,16,17,18]. The slight reduction of OTA between green coffee and roasted coffeecould be due to three events: physical removal of OTA with chaff [19], isomerization at the C3 position into a less toxic diastereomer [15] and thermal degradation of OTA with possible involvement of moisture [15,19,20,21]. The presence of OTA in coffee is undesirable due to its toxicity and that it may serve as a barrier to trade affecting the economies of coffee producing countries. Thus, OTA levels usually need to be tested and guaranteed to be below certain levels. Many countries have established maximum limits for OTA that can be present in food. The legislation in Europe is 5 µg/kg of roasted coffee [23]. 

Therefore, it is essential to use reliable and sensitive analytical methods for the detection of OTA in individual foodstuff. There are numerous methods for the determination of OTA in foodstuffs, such as thin layer chromatography, enzyme-linked immunosorbent assay (ELISA), high performance liquid chromatography (HPLC) and liquid chromatography-mass spectroscopy (LC-MS) [24]. The analysis of OTA in coffee is complex due to interfering colored substances. Two methods have been validated in Europe for analyzing OTA in coffee: one is based on acidic extraction and a partition method for cleanup [25] and the second one uses immunoaffinity column (IAC) [26]. The aim of this study was to analyze several samples of ground coffee sold in the French supermarket and to evaluate the OTA intake via beverage. For this purpose, 30 coffee samples were purchased in French supermarkets and OTA was analyzed using both the official methods [25,26]. By comparing the data obtain with both methods and establishing calibration curves, it appeared that for low amounts of OTA (<2 µg/kg), almost all OTA was lost when performing the alkaline method [25]. For this reason, for some samples we tested the method described in 1996 by Pittet *et al.* [27] in which the IAC is performed at pH 7.

## 2. Experimental

### 2.1. Chemicals

All reagents (potassium chloride, sodium hydrogen carbonate, sulfuric acid, phosphoric acid, hydrochloric acid, acetic acid, sodium dihydrogen phosphate) were of normapur grade. All solvents (methanol, chloroform, acetonitrile, propan2-ol, n-hexane) were of HPLC grade from ICS (France). Deionized water was used for the preparation of all aqueous solutions and for HPLC. OTA and OTB standards, free from benzene and carboxypeptidase were from Sigma Chemicals (France). The immunoaffinity columns (IAC) (Ochraprep®) were from Rhone Diagnostic technologies (France). OP-OTA was prepared as described by Xiao *et al.* 1996 [28]. In brief, 1 mg of OTA was dissolved in 300 µL of DMSO. Then 300 µL of NaOH (1N) were added. The mixture was incubated at room temperature for 24 h. This time is long enough to convert all OTA into OP-OTA. OP-OTA was analyzed by HPLC with fluorimetry detection. The structure has been identified by LC-MS/MS.

### 2.2. Extraction of OTA from roasted coffee

Thirty ground roasted coffee samples were purchased in French supermarkets. OTA was extracted by three methods: two using IAC cleanup [26,27] and the third using toluene extraction under acidic conditions [25]. The different methods are detailed below.

#### 2.2.1. Extraction of OTA in acidic conditions according to the EU validated method “15141-1” [25]

Twenty grams of ground coffee were mixed with 30 mL HCl 2 M; 50 mL MgCl_2_ 0.4 M and 100 mL Toluene. The mixture was continuously shaken for 60 min. After separation, 50 mL of toluene phase containing OTA was purified on SEP pack. The column was washed twice with 10 mL n-hexane, twice with 10 mL acetone/toluene (5/95, v/v), and once with 5 mL toluene. OTA was recovered by addition of 15 mL toluene/acetic acid (90/10, v/v) two times. 30 mL of 5% natrium bicarbonate solution was added twice. The mixture was shaken for 10 min. After separation, OTA was present in the aqueous phase. The solution was acidified by the addition of concentrated HCl until pH 2 was reached. Finally, 60 mL of CHCl_3_ was added and the mixture was shaken for 10 min. After separation, the chloroformic phase was recovered. This step was performed twice. Chloroform containing OTA was evaporated to dryness. OTA was dissolved in methanol (500 µL). 

#### 2.2.2. Extraction of OTA in alkaline conditions according to the EU validated method “14132” [26]

Ten grams of ground coffee were mixed with 100 mL of solution containing methanol/natrium bicarbonate 3%, 50/50 (v/v) and shaken for 30 min. The solution was filtered through Whatman paper No. 4. Twenty milliliters of this extract were diluted with 20 mL of natrium bicarbonate 3% and purified on a phenyl silane column. The column was washed with 10 mL methanol/natrium bicarbonate 3%, (20/80, v/v) and 5 mL of natrium bicarbonate 1%. OTA was recovered by addition of 10 mL methanol/water (7/93, v/v). 30 mL of phosphate buffer solution (PBS) (containing 8 g NaCl, 1.2 g di-natrium hydrogeno phosphate (Na_2_HPO_4_), 0.2 g kalium dihydrogenophosphate (KH_2_PO_4_), 0.2 g KCl, and 900 mL water) were added to 10 mL of extract. This mixture was purified on an Ochrastar Immuno Affinity Column (IAC). The column was washed twice with 10 mL PBS. OTA was recovered by elution of 1.5 mL of methanol, evaporated to dryness and dissolved again in 500 µL methanol.

#### 2.2.3. Extraction of OTA according to Pittet *et al.* 1996 [27]

Twenty five grams of ground coffee were mixed with 200 mL methanol/natrium bicarbonate 3%, 50/50 (v/v) and shaken for 10 min. The solution was filtered through Whatman paper No. 4. Four milliliters of this extract were mixed with 100 mL PBS. This mixture was purified on an IAC. The column was washed two times with 10 mL ultra pure water. OTA was recovered by elution in 4 mL of methanol, evaporated to dryness and dissolved again in 500 µL methanol.

### 2.3. Preparation of coffee beverage and extraction of OTA

**Figure 1:**
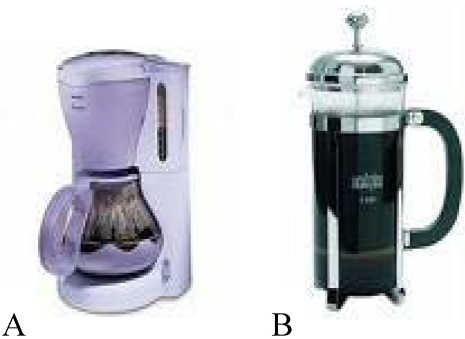
Coffee makers: (**A**) electric coffee maker (filtration system), and (**B**) piston machine.

OTA was also analyzed in coffee beverages that were made using two different kinds of machines (filtration technique and piston) (Figure 1). Using the electric machine (filtration technique), 25 g of coffee were put on the filter and slowly extracted with 300 mL hot water. In the second method, 30 g of coffee were placed in the container, and then brewed for 5 min in the presence of 300 mL of boiling water. The resulting beverage was used as the extract for OTA analysis. Purification was done on an IAC using the conditions described in Section 2.2.2 (Entwisle’s method [26]). In brief, 75 mL of each OTA extract (corresponding to 7.5 g of coffee with electric machine and 9 g with piston machine) were diluted with 25 mL phosphate buffer solution containing 24 g of NaCl, 3.6 g di-natrium hydrogeno phosphate (Na_2_HPO_4_), 0.6 kalium dihydrogenophosphate (KH_2_PO_4_), 0.6 g KCl, 900 mL water adjusted to pH 7.4 and loaded on the IAC. A second purification was made according the Pittet’s method [29]. In this case, 20 mL of extract were diluted with 80 mL PBS and loaded on IAC. In each case the IAC was washed with 20 mL PBS, followed by OTA elution using conditions analogous to OTA extraction from ground coffee extract.

OTA has also been extracted with cold water. Thirty grams of coffee were placed in the presence of cold water for 24 h. In each case, the beverage (20 mL) was used as extract and diluted with 80 mL of PBS then passed through the IAC.

### 2.4. Detection of OTA and quantification

OTA was analyzed by HPLC using C18 column PRONTOSIL 120 (250 × 4.0 mm^2^) with inner porosity of 3 µm, with an ultrasep C18 10 µm precolumn, 1 cm long from ICS, under isocratic condition (mobile phase: orthophosphoric acid at 0.33 M/ACN/propan-2-ol (600:400:55), flow rate 0.8 mL/min). HPLC analysis used a Gilson 811B dynamic chromatography pump coupled to a Spectra Physics 2000 ﬂuorescence spectrophotometer and an ICS autosampler. Detection was performed with a programmable Merck HITACHI FL Detector L-7485 (excitation 340 nm, emission 465 nm). The chromatograms were analyzed by Normasoft software provided by ICS (France).

OTA standard solutions were prepared by dissolving 10 mg of OTA in 1 mL of methanol. A series of working standards from 0.2 to 100 ng OTA was prepared by dilution (equivalent to 0.02 to 15 ng/g of OTA in coffee). They were used to calibrate the LC detector response. The concentration of the OTA stock solutions was determined by measuring the UV absorbance at 333 nm with a molar extinction coeﬃcient ε of 5500 mol^−1^cm^−1^. The quantity of OTA contained in the extract was evaluated by integration of the peak area corresponding to OTA. Depending of the method of extraction, 500 µL of methanol correspond to 10 g, 2 g or 0.5 g of coffee, respectively, after acidic extraction [25], alkaline extraction [26], and Pittet’s method [27].

The coeﬃcient of linearity (R^2^) was 0.997. The limit of detection (LOD) was 0.025 µg/kg and the limit of quantification (LOQ) was 0.04 µg/kg at nine times background.

OTA recoveries: Three coffee samples spiked with 5 µg/kg (corresponding to EU legislation), were extracted by the three methods and were analyzed on the same day, by the same operator and with the same HPLC system. The average recoveries were 45 ± 4% for Entwisle method [26]; 71 ± 1% for Pittet method [27] and 57 ± 0.5% for acidic method [25]. When beverages were used as extract, the average recovery was 80 ± 0.5%. Repeatability test for OTA analysis: The three coffee samples spiked with 5 µg/kg (corresponding to EU legislation) were extracted by the three methods and were analyzed on five successive days by the same operator and with the same HPLC system. The average OTA concentration was 2.5 ± 0.20 µg/kg for the Entwisle method [26]; 3.6 ± 0.3 µg for Pittet method [27] and 3 ± 0.3 µg for acidic method [25]. When beverages were used as extract, the OTA concentration was 4 ± 0.1 µg.

### 2.5. Confirmation of OTA derivatives

The confirmation of the presence of OTA (or OTA derivatives) in coffee samples was achieved by the following technique: an aliquot, taken from the purified extract of a sample where OTA was detected by the HPLC analysis, was dried. The pellet was dissolved in 975 µL of a buffer solution of 0.04 M Tris-HCl, 1 M NaCl, pH 7.5. Then, 25 µL of carboxypeptidase (100 U/mL H_2_O) was added and the mixture was incubated at room temperature overnight. The sample was analyzed under the same HPLC chromatographic conditions as used above. The OTA peak disappeared whereas the peak of α-OT appeared. The OTB peak disappeared whereas the peak of β-OT appeared. 

## 3. Results and Discussion

### 3.1. Occurrence of OTA in ground roasted coffee

All coffee samples were analyzed for OTA content using both official methods [25,26,29]. Some coffee samples were also analyzed by the method published by Pittet *et al.* [27]. Before conducting the determination of OTA occurrence in the samples, the recoveries using these methods was evaluated.

#### 3.1.1. Comparison of the recovery of OTA using the three methods

Ground coffee (10 different samples) was spiked with increasing amounts of OTA (final concentration in coffee equivalent to 0.5, 1, 2.5, 5, 10 µg/kg). Table 1 shows the % recoveries (all the analyses were done in duplicate). 

**Table 1:** Comparison of the recoveries in ground coffee.

Contamination level analysis method	0.5	1.0	2.0	2.5	5.0	10.0 µg/kg
	% recovery					
EN No. 14132 [26]	16.6–18.7	24.5–27.0		33.0–34.0	41.0–49.0	63.0–70.0
Pittet method [27]		60–64	62–65		70–72	80–82
EN No.15141-1 [25]	50–55	53–55		57–55	57–57	

The recovery limits commonly accepted for a level of contamination of OTA around 10 µg/kg are 70–125% [30]. For this concentration of OTA, the % recovery with both IAC methods were between these two limits; however, the % recovery for lower concentrations of OTA from roasted coffee was lower than 70% with Entwisle’s method [26]. The recovery by Entwisle’s method increased with the amount of OTA in roasted coffee beans and could be considered as acceptable only for amounts >5 µg/kg. The recovery by the Pittet’s method [29] is relatively constant (an average of 70% for all amounts of OTA). The recovery after toluene extraction in acidic condition (EN 15141-1 [24]) was constant, but is not greater than 60%. In this later case, the loss is mainly due to the difficulty to evaporate toluene to dryness, forcing the addition of chloroform extraction. Horwitz [30] recommends that recoveries less than 60–70% may be subject to investigations leading to improvement.

The major difference between the two IAC methods was the relative proportion of PBS added to the extract and the duration of shaking incubation. The conditions in Pittet’s method [29] bring the pH much closer to neutrality than the Entwisle’s condition where the pH remains above 8. pH conditions that are too high lead to the formation of open-ring OTA (OP-OA), which is not recognized by the OTA antibodies. This phenomenon has already been observed during breakfast cereal analysis for OTA [31,32,33,34]. The large dilution of the extract in the Pittet’s method [29] before IAC columns lowers the pH in a range allowing reconversion of almost all OP-OTA into OTA before IAC, which is not the case in the Entwisle’s method where almost all OTA remains in OP-OA form.

#### 3.1.2. OTA amount in ground roasted coffee

Thirty samples were analyzed by both official methods [24]. Ten samples were analyzed by the method of Pittet [29] (Table 2). 

In all samples, trace amounts of OTA (<LOD) could be detected. The amount of OTA, whatever the method of extraction, ranged from trace (<LOD) to 11.9 µg/kg. For 27% of the samples, the OTA amount ranged between LOD and 0.5 µg/kg, 33% samples contained OTA between 0.5 and 1 µg/kg, while 37% samples contained OTA between 1 and 3 µg/kg. Only one sample (3%) contained OTA above the EU limit of 5 µg/kg [23] and reaches a very high level of 11.9 µg/kg with the EN 14132 method [26]; 15 µg/kg with the EN 15141-1 [25] method and 10 µg/kg with the Pittet’s method [27]. This highest contamination was found in a sample without trademark.

For six samples out of 10 analyzed, the amount found by Pittet’s method [27] was significantly higher than with Entwisle’s method [26]. As mentioned in the previous paragraph, the main explanation is the reconversion of OP-OA into OTA after large dilution of extract in the Pittet’s method. A second explanation is the lowest amount of interfering compounds with OTA antibodies. Indeed, the amount of coffee extract passed through the column, by the method of Pittet *et al.* [27] corresponds to 0.5 g of coffee and by that of Entwisle *et al.* [25] corresponds to 2 g. Therefore, the amount of compound possibly interfering with the OTA antibody is four-fold greater using this later method. All these experiments were run in parallel with the same batches of coffee, the compounds extracted are expected to be identical and in a similar ratio. 

In 9 samples (#9, 19, 20, 21, 22, 24, 27, 28, and 29), OTB (dechlorinated OTA) was detected, but not quantified. OTB could lead to underestimation of OTA (see paragraph 3.3).

**Table 2:** Occurrence of OTA in ground coffee (µg/kg) as determined by three different extraction and clean-up methods (CEN14132, CEN 15141-1, Pittet method). Data are expressed as µg/kg without taking recovery into account. NA: not analyzed; LOD (limit of detection): 0.025 µg/kg; LOQ (limit of quantification): 0.04 µg/kg.

Sample	CEN14132 (acidic condition)	CEN 15141-1 Entwilse, 2001	Pittet method
1	1.6	1	1.34
2	0.8	1.6	2
3	0.68	0.66	*NA*
4	1.1	0.5	0.65
5	0.4	0.5	*NA*
6	1.3	0.5	*NA*
7	1.3	1	*NA*
8	1	1	*NA*
9	1.05	1.35	1.3
10	LOQ	LOQ	*NA*
11	LOQ	LOQ	*NA*
12	0.93	0.5	*NA*
13	0.5	0.35	*NA*
14	LOD	LOD	*LOD*
15	0.8	0.5	*NA*
16	0.9	0.83	1.03
17	0.41	0.35	*ND*
18	1.69	1.25	1.41
19	0.6	0.5	*NA*
20	0.75	0.5	*NA*
21	0.5	0.5	*NA*
22	0.87	0.75	1.1
23	0.75	0.92	*NA*
24	11.89	15.08	10.14
25	LOQ	LOQ	*ND*
26	LOQ	LOQ	*ND*
27	0.76	0.75	1.2
28	1.37	1.25	1.6
29	0.7	0.5	*NA*
30	0.54	0.35	*NA*

Our results are in good agreement with the findings of others. For example, reported amounts of OTA in coffee have been from 0.5 to 23.0 ng/g [35], 9.9 to 46 ng/g [14], 0.2 to 5.5 ng/g [8], 0.1 to 17.4 ng/g [9], 0.1 to 4.6 ng/g [10], and 0 to 48 ng/g [11]. An average contamination of 2.4 ng/g was found in 50 coffee bean samples [36]. Of the 40 samples analyzed, only five were contaminated with OTA in concentrations that varied from 0.64 to 4.14 ng/g, with an average in the positive samples of 2.45 ng/g [37]. Pardo *et al.* (2004) [38] analyzed 14 green coffee samples of Asian origin and reported OTA concentrations ranging from 1.6 to 31.5 ng/g with an overall mean of 6.0 ± 7.9 ng/g. In the study of Gopinandhan *et al.* (2007) [39], a total of 129 green coffee samples, randomly collected from curers, traders and local auction platforms, were tested for OTA. The results indicated that 81% of samples had OTA levels below 5 ng/g. Only three samples had OTA levels above 5 ng/g and the highest level of OTA was 11.7 ng/g in a robusta cherry sample. Analysis of 80 green coffee samples from India indicated that, although a high incidence (74%) of OTA contamination (0.2–13.5 ng/g) was recorded, the overall mean OTA level (2.17–2.45 ng/g) was low. The highest recorded OTA concentration was 13.5 ng/g in a robusta cherry sample and only five samples had OTA levels above 5 ng/g. The mean OTA level was higher in cherry (range: 1.63 ± 0.97 to 4.8 ± 3.90) than parchment (0.56 ± 0.35–1.10 ± 0.28), indicating a correlation between processing method and OTA contamination [40].

Post-harvest practices, particularly drying, affect OTA contamination [41]. The highest risk of exposure to contamination was characterized by fruit contact with the soil, constituted by the fraction coffee swept from ground, and by inadequate post-harvest handling of the product during drying in ground coffee yards [42].

### 3.2. OTA in the beverage

#### 3.2.1. Recovery of OTA

To analyze the amount of OTA passing through the beverage, only clean-up on IAC without extraction was performed. Coffee beverage is prepared by passing hot water over ground coffee beans and is equal to the crude extract. The recovery of OTA was evaluated on coffee spiked with increasing amounts of OTA before infusion, or by addition of OTA to the crude extract. In this analysis the % recovery of OTA was fairly constant (Table 3), because OTA is not treated with base (leading to generation of OP-OA) prior to extraction from the beverage. 

**Table 3:** Percent of recovery from coffee beverage used as extract.

Contamination level analysis method	0.5	1.0	2.0	2.5	5.0	10.0
	% recovery					
Entwisle 14132	68–72	75–73		72–70	80–73	68–70
Pittet		72–70		75–76	80–80	

#### 3.2.2. Amount of OTA in the beverage

The most contaminated roasted coffees were used to prepare coffee beverage. Nineteen samples were used to prepare beverages using two different techniques: the filtration technique (infusion) or piston technique (decoction). The percentage of OTA passing through the beverage was calculated using the value obtained by the EN 14132 method [25] as 100% value in ground coffee (bold value in the Table 4) or using the value of Pittet’s method [27] as 100% value in ground coffee (italic value in the Table 4). 

The amounts of OTA passing through the beverage are slightly higher (ranging from 5–20%) when the coffee is made by the electric machine (infusion) compared to piston machine (decoction).

The percentage of OTA found in the beverages varied from 13–141% of the initial amount of OTA found in ground coffee (Table 4). 

Several authors described also that OTA present in roasted coffee essentially passes completely into the brew (or found even more OTA), whereas some others did not find any passage [8,14,15,16,36,43]. Mocha brewing, espresso and Scandinavian boiled coffee allow passage of more OTA than the other mode of coffee preparation such infusion.

Percentage of OTA passage over 100% confirms the presence of masked OTA, probably isomerization of OTA by roasting [21,43] and interference of some compounds extracted from the ground coffee and the antibodies. OTB is one of the interfering substances (see paragraph 3.3). Indeed, OTB was found in all coffee beverages made with ground coffee samples in which more than 0.8 µg/kg OTA were detected. OTA analysis from beverage do not include alkalinization step before IAC and thus no OP-OA is formed. 

**Table 4:** Amount of OTA passing through the beverage.

code	Mode of preparation	ng/L coffee	ng/kg coffee	% passing *
1	electric	22	0.22	**13/***16*
	piston	32	0.25	**15/***19*
2	electric	47.2	0.48	**60**
	piston	37.93	0.38	**47.5**
4	electric	72.27	0.72	**65/***110*
	piston	58.75	0.46	**42/***71*
6	electric	31.18	0.31	**24**
	piston	32.6	0.25	**19**
7	electric	26.37	0.26	**20**
8	electric	20.68	0.21	**21**
9	electric	148.55	1.48	**141/***117*
	piston	141.93	1.11	**110/***88*
12	electric	LOD	LOD	-
14	electric	48	0.48	**>100**
	piston	43.6	0.34	**>100**
15	electric	22.42	0.22	**27.5**
16	electric	79.63	0.8	**86**/*78*
	piston	85.93	0.67	**69**/*65*
17	electric	LOD	LOD	<LOQ
18	electric	122.78	1.23	**73**/*87*
	piston	168.65	1.32	**78**/*94*
19	electric	11.87	0.12	**20**
22	electric	77.66	0.78	**87**/*74*
	piston	79.88	0.62	**69**/*59*
24	electric	796.03	7.96	**67**/*78*
	piston	882.31	6.89	**58/***68*
27	piston	27	0.27	**35.5/***22*
28	electric	161.28	1.6	**117/***100*
	piston	170.93	1.34	**98/***84*
29	electric	31	0.31	**44**

* Bold values give % of OTA calculated taking into account the OTA measured in ground coffee using EN 14132 method [25];. Italic values correspond to the % of OTA in coffee found using Pittet’s method [29]; in both ground coffee and beverage.

### 3.3. Interference of OTB and other compounds with OTA antibodies and modification of the structure of OTA during extraction explaining underestimation of OTA content in ground coffee

As in the case of breakfast cereal [32,33], some compounds interfered with OTA during IAC cleanup. In the most contaminated coffee, the highest amount of OTA was obtained using the method EN 1514-1 [24], which does not use purification on IAC and moreover is based on extraction after acidification.

**Figure 2:**
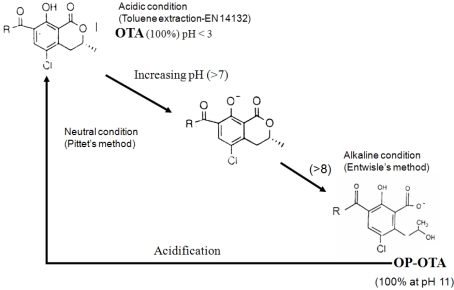
Ring opening formation of OTA (OP-OA) under alkaline condition.

Formation of OP-OA by alkalinization leads to the underestimation of OTA. This loss is partially counteracted in the Pittet’s method because the large dilution of the extract lowers the pH, allowing reconversion of OP-OA into OTA (Figure 2). 

Interference is also due to the presence of OTB, which was detected in the most OTA contaminated coffee.

For this reason, we tested the cross reactivity of OTA, OTB and OP-OTA with the antibodies from the IAC used. When 50 ng of each toxin is spiked on a coffee extract before IAC, the recoveries of OTA and OTB were respectively of 57% and 81%, whereas OP-OTA is not recognized (amount detected is below the LOQ). The respective recoveries when each derivative is spiked independently were 80% for OTA, 91% for OTB and 0% for OP-OTA. This indicates that: (1) OTB is recognized by the antibodies of the OTA IAC; (2) the affinity of OTB is higher than that of OTA on these columns; (3) OP-OTA is not recognized by OTA-IAC. 

This explains the data obtained with the most contaminated coffee sample, for which much higher OTA amounts are found in acidic conditions without IAC (15 µg/kg) than by the two other methods (9.5 µg/kg). Thus, the loss of OTA by the two methods—including IAC cleanup—is due to the transformation of OTA into OP-OTA and the presence of OTB, which has a higher affinity and contribute to saturation of column. 

## 4. Conclusions

Our data show that OTA in coffee is generally underestimated due to OTB and other substances also present in coffee that interfere with the OTA antibodies. In addition, extractions in alkaline medium lead to conversion of OTA into open-ring OTA (OP-OA), which is not recognized by OTA antibodies. In general, the amount of OTA found in ground coffee was below the limit of residue enforced by EU [23]. Nevertheless, OTA intake via coffee beverage is not negligible in view of its potential carcinogenic properties (for a review see Pfohl-Leszkowicz and Manderville, 2007 [4]). In several countries (France, Italy, Sweden, Spain, and Portugal, for example), the coffee beverage is made with 7 g of ground coffee for 100 mL of water, whereas in others (USA, for example), only 4 g of coffee/100 mL of water are used. The highest amount of OTA found in ground coffee was 11.9 µg/kg. Assuming a total transfer of OTA from ground coffee to beverage, a bowl of 300 mL of coffee made with 7 g will contain 250 ng of OTA; a cup of expresso (85 mL) will contain 70 ng. Thus, the intake of a high coffee consumer who drinks 640 mL of coffee (1 bowl of 300 mL at breakfast; 1 cup of 85 mL at 10 o’clock; 1 cup after lunch; 1 cup in the afternoon; 1 cup after dinner) will reach 530 ng. For a human weighing 60 kg, this intake via coffee corresponds to half of the tolerable daily intake of OTA established by JECFA (joint expert committee of Food and Additives) [43]. This intake of 530 ng is six times higher than the virtual safety dose (VSD) (1.5 ng/kg bw/day) established on the kidney tumors [44]! If we make the same calculation using the average OTA content (1.5 µg/kg) found in the coffee, the amount of OTA in a bowl is 31 ng; and in a cup of expresso 8.8 ng. Thus a heavy consumer will have a daily intake of 66.2 ng/day equivalent to 1.1 ng/kg bw/day, which is close to the VSD. It should be kept in mind that excessive coffee consumption (more than three cups a day) was implicated in upper tract transitional cell carcinoma and bladder cancer [45,46]. The risk is higher for heavy coffee drinkers carrying the genotype GSTP1 105–104 val. [47] and can be explained by the fact that glutathione conjugation is involved in the biotransformation of OTA into genotoxic and carcinogenic products [48,49,50] leading to DNA adducts [51,52]. As the percentage of OTA passing through the beverage depends on the origin and the roasting process of coffee, the intake evaluation should be done on the amount of OTA found in the beverage. A simple method to evaluate the amount of OTA in coffee could be to extract OTA with hot water. This extract could then be directly used on the IAC column. 
